# SCL-90 empirical factors predict post-surgery weight loss in bariatric patients over longer time periods

**DOI:** 10.1007/s40519-022-01424-4

**Published:** 2022-07-13

**Authors:** Umberto Albert, Tommaso Bonavigo, Oriana Moro, Elide Francesca De Caro, Silvia Palmisano, Elisabetta Pascolo-Fabrici, Federico Sandri, Nicolò de Manzini, Lisa Di Blas

**Affiliations:** 1 Department of Medicine, Surgery and Health Sciences, University of Trieste, Trieste, Italy; 2 Department of Mental Health, Azienda Sanitaria Giuliano-Isontina-ASUGI, Trieste, Italy; 3 Department of Life Sciences, University of Trieste, Via Weiss 2, 34100 Trieste, Italy; 4 IRCCS Materno Infantile Burlo Garofolo, Trieste, Italy

**Keywords:** SCL-90, Bariatric surgery, Longitudinal data, Protective factors, Factor analysis, Obesity

## Abstract

**Purpose:**

This longitudinal study examined how pre-intervention psychological health helps predict bariatric surgery (BS) success as percentage of expected body mass index loss (%EBMIL) over shorter to longer periods.

**Methods:**

Adult candidates for BS (*N* = 334, 67.4% females) completed the Symptoms Checklist 90 (SCL-90) questionnaire; on average, 11 months occurred between the pre-surgery psychological evaluations and the bariatric intervention. We explored the factor structure of the SCL-90 items and inspected how SCL-90 empirical factors compared with SCL-90 scales and general indices predicted %EBMIL at 3–6-month, 1-year, and 2-year follow-up occasions, adjusting for gender, pre-intervention use of antidepressants and actual and ideal BMIs.

**Results:**

Factor analysis combined the 90 items into 8 factors, which partially replicated the expected item structure. The SCL-90 empirical factors (but not the SCL-90 scales and indices) contributed to predict BS success. In fact, the Relational Distress factor directly protected from weight regain at 1-year follow-up, indirectly via 1-year %EBMIL at the 2-year follow-up, when it further strengthened the impact of the empirical factor of Generalized Anxiety on the 2-year BS outcome. The results also evidenced a cascade effect of the pre-surgery actual BMI across time as well as unique and direct effects of pre-surgery use of antidepressants and perceived ideal BMI on the 2-year outcome.

**Conclusions:**

SCL-90 empirical factor scores for obese patients are more efficient in anticipating BS success compared with original scale scores. They reveal that relational distress and anxiety are risk factors for postoperative weight loss, in addition to pre-intervention actual BMI, antidepressant therapy, and perceived ideal BMI.

**Level of evidence:**

III, well-designed cohort.

## Introduction

Obesity and mental health are two global public health problems that are usually linked. Although findings are not entirely consistent, the research literature shows that concurrent associations between obesity and dysregulation of mood, thoughts, and behaviors are not spurious [1, 2]. Longitudinally, obesity and psychological problems are mutually dependent [3, 4]. Accordingly, in clinical practice psychopathological health problems are not uncommon in obese candidates for bariatric surgery (BS) [5, 6]. In fact, the criteria for selection of BS candidates depend on their mental health as well as their physical condition. However, the influence of pre-intervention psychological problems on the success of BS in terms of weight loss is not clear yet and under investigation [7].

Current literature shows that before surgery bariatric candidates generally exhibit higher emotional instability and dysregulation, lower interoceptive awareness and self-esteem, higher levels of addiction and disordered eating behaviors, if compared with non-clinical samples [8–10]. Furthermore, pre-intervention patients, particularly if they are binge-eaters, tend to report higher levels of general psychopathological symptomatology and mood and anxiety-related disorders compared with non-clinical samples [6, 11, 12], while female candidates generally score higher on global symptomatology as well as on domains, such as depression, somatization, and interpersonal sensitivity [10, 13]. Accordingly, studies report that use of a psychotropic medication is more likely among obese and bariatric patients compared with people with weight in the range of normal weight. Antidepressants especially are used by both male and female patients [14], with obese men being particularly at risk compared with men of normal weight [15].

Findings instead are still inconsistent, when the impact of pre-intervention psychological risk factors on post-intervention outcomes is investigated. For example, in some studies depression and binge eating predicted a worse postoperative outcome, but in other studies they did not account for postoperative weight loss or even protected against weight regain [11]. Similarly, longitudinal results on the Symptoms Check List (SCL-90) [16] a screening self-report questionnaire applied to assess psychopathology [17, 18] have suggested a predictive effect of general symptomatology, i.e., the SCL-90 General Severity Index, on bariatric surgery success [19, 20], but inconsistent findings for specific clusters of psychopathological symptoms. Indeed, in a small sample of bariatric patients, Brunault et al. [21] found that preoperative phobic anxiety, interpersonal sensitivity, and depression were (marginally) responsible for postoperative weight loss 1 year later. However, Ortega et al. [22] found that preoperative self-esteem and body image but not psychopathology were predictors of postoperative outcomes. Not even objective indicators of psychological unhealthiness, such as psychotropic medication use, including antidepressants, affects post-surgery weight loss, within 12 months [23, 24].

Used worldwide in the general population as well as in clinical populations, such as obese patients, the SCL-90 questionnaire has been demonstrated to psychometrically satisfy reliability and diagnostic validity in BS candidates [18, 25]. Nevertheless, no empirical study has attempted to examine its factor structure in obese patients, although results on the replicability of the SCL-90 factor structure have been inconsistent in both non-clinical and clinical samples, and consequently several shorter and less comprehensive versions have been developed [26]. For example, Olsen et al. [27] reported that the SCL-90 Psychoticism subscale was problematic in a Danish sample, while Prunas et al. [28] opted for an 8-component solution and showed that the eight dimensions partially recombined the items into conceptually coherent domains, such as relational distress or general dysphoria. In clinical samples, the expected factor structure was replicated neither, and the SCL-90 questionnaire was recommended more as a screening instrument for general distress to be supplemented by other diagnostic instruments [29, but see 30].

Finally, empirical research has systematically examined the impact of preoperative actual weight or body mass index (BMI) on postoperative success, with results often indicating a negative association [31]. Conversely, less attention has been paid on a BS candidate’s perceived ideal weight and its impact on post-surgery weight loss, although an individual’s desired or ideal body image plays a significant role in eating disturbances [32]. The currently available findings suggest that before intervention obese patients tend to refer postoperative ideal body silhouettes, which are generally larger compared with the normative target figures [33]. Such a tendency to report inflated ideal postoperative weight, that is, overestimating an ideal target healthy weight, is hypothesized to obstacle an optimal weight loss after bariatric surgery [34], but to our knowledge no empirical results on the impact of a BS patient’s ideal target weight on her/his weight loss are available.

In the current 2-year follow-up study, we aimed at contributing to understand how pre-surgery psychopathological conditions predict post-surgery weight change. Specifically, we investigated the extent to which preoperative mental health as SCL-90 scores helps predict the success of bariatric surgery 3–6, 12, and 24 months after surgery. To this aim, we examined the SCL-90 factor structure in our sample of obese patients and explored whether an alternative item organization could improve prediction compared with the original SCL-90 scale scores. In addition, we examined how the use of antidepressants and anxiolytics and a patient's perception of her/his ideal body weight affects post-surgery weight loss. Finally, we developed a set of SCL-90 normative scores for bariatric candidates: A raw cutoff score of 0.9 is generally considered in clinical practice, relevant regardless of gender [35], but no normative data are available for bariatric candidates.

## Methods

### Participants

At interview (t0_INT_), participants were 334 obese patients (67.4%women), all of which were clinically eligible for bariatric surgery after a thorough clinical objective evaluation procedure (see Procedure), at Trieste University Hospital, between January 2011 and December 2019; their mean age was 42.1 ± 10.9 (18–65) and their mean BMI kg/m^2^ 42.2 ± 6.9 (29.4–73.0), with men (*M* = 43.7) having slightly higher BMI values than women (*M* = 41.5, p ≤ 0.05, *η*^2^ = 0.02). Among these candidates, 193 underwent bariatric surgery (111 gastric bypass, *M*_BMI_ = 42.2 ± 4.5; 82 sleeve gastrectomy *M*_BMI_ = 45.5 ± 7.8), whereas 53 were inserted an intragastric balloon (*M*_BMI_ = 36.6 ± 4.8). Among the remaining 88 eligible patients, 66 voluntarily decided not to continue the therapeutic path, and 16 were on the waiting list for surgery; for 6 patients post-interview data were incomplete. No differences in mean BMI (*p* > 0.10) emerged between candidates who underwent surgery and those who did not.

BS patients were evaluated 3–6 months (*N* = 157), 12 months (*N* = 166) and 24 months (*N* = 102) after surgery.

### Procedure and treatments

Routinely, at Trieste University Hospital, obese patients who are candidates for bariatric treatments are selected after an extensive preoperative assessment by a multidisciplinary team, including bariatric surgeons, dieticians, gastroenterologists, psychologists, and psychiatrists. Inclusion criteria for bariatric surgery are in accordance with the International Guidelines for the evaluation of candidates for bariatric surgery [36]: Well-informed patients, with acceptable operative risks, previous failures of non-surgical treatments, declared compliance to follow lifelong medical surveillance, age between 18 and 65 years, BMI ≥ 40 kg/m^2^ or between 35 and 40 kg/m^2^ in presence of obesity-related comorbidities. Exclusion criteria include the presence of a current mental disorder (e.g., bipolar disorder or schizophrenia not compensated, or substance use disorder) or a current diagnosis of Bulimia Nervosa or Binge Eating disorder not compensated and not treated. Eligible patients included in the present study were free from any current psychiatric condition which could represent a contraindication for bariatric surgery.

Surgical interventions on the present sample of patients were performed laparoscopically. Though technically different, both sleeve gastrectomy and gastric bypass are aimed at permanently restricting the amount of food a stomach can hold and changing the way stomach and intestine absorb and digest food; these techniques have an impact on metabolism as well. Overall, they represent the most common procedures worldwide and are very effective in term of weight loss and weight-related comorbidities resolutions [36]. Therefore, they were primarily performed in Trieste over other surgical techniques. More in detail, the choice for the most appropriate intervention for each patient is based on clinical issues. Notably, if the preoperative gastroscopy shows signs of gastroesophageal reflux, the surgeon will suggest laparoscopic gastric bypass, since the reflux disease may worsen after sleeve gastrectomy operation for intraoperative technical reasons; but if the patient has life-saving oral therapy, surgeons will opt for sleeve gastrectomy. An intragastric balloon instead is a temporary non-surgical treatment which is removed within 6 months. Therefore, selection criteria for surgical vs. endoscopic treatments are different and also clinical results of the two treatments (weight loss and commitment) generally are different [36, 37].

### Ethical statement

All candidates were adults and voluntarily agreed on the assessment procedure. In general, all patients who present to our outpatient service sign a written informed consent, approved by the Ethics Committee at Trieste University Hospital, that they provide clinical data which could be used for teaching and research purposes, provided that these data are anonymously treated. Moreover, before surgery, all eligible patients were well informed about the surgical technique, surgical risks, and postoperative complications, and surgeons ensured that patients understood the information received and signed a full informed consent for the operation. All ethical guidelines required for the conduct of research involving human subjects were followed, including compliance with the legal requirements in Italy (UE, 2016/679, D. Lgs.196/2003, D.Lgs.101/2018).

### Measures

#### Symptom checklist-90

SCL-90 is a self-report questionnaire designed to assess psychopathological conditions in a general population. Several versions are available [25] and we administered the SCL-90 version [16]. Patients reported their own behaviors, feelings, and thoughts as they had occurred in the past week, on a 5-point Likert scale. SCL-90 assesses 10 psychopathological scales and provides 3 global indices (Table 1), among which The Global Severity Index (GSI), a screening index of overall distress. Zero-order correlations among SCL-90 scales ranged from 0.40 to 0.81; internal consistency was high (Table 1).

**Table 1:** SCL-90 scales and global indices: Alpha reliabilities, descriptive statistics, and clinically relevant scores for bariatric female and male candidates

	Alpha	*M*	(SD)	Women			*M*	(SD)	Men			*F*
				Percentile ≥					Percentile ≥			
				80	90	95			80	90	95	
Somatization	0.88	1.07	(0.74)	1.7	2.2	2.6	0.82	(0.67)	1.3	1.6	2.5	8.04**
Obsession–Compulsion	0.86	0.71	(0.66)	1.2	1.7	2.2	0.63	(0.63)	1.2	1.5	1.7	0.90
Depression	0.90	0.86	(0.74)	1.4	2.0	2.3	0.61	(0.65)	1.1	1.5	2.1	8.82**
Anxiety	0.87	0.60	(0.61)	1.0	1.4	1.9	0.50	(0.77)	0.6	1.1	1.2	1.99
Phobic Anxiety	0.78	0.32	(0.52)	0.7	1.0	1.4	0.16	(0.37)	0.3	0.6	0.8	7.20**
Psychoticism	0.85	0.44	(0.57)	0.7	1.2	1.4	0.36	(0.49)	0.6	1.1	1.3	1.36
Interpersonal Sensitivity	0.87	0.83	(0.81)	1.5	2.0	2.1	0.59	(0.60)	1.2	1.6	1.8	7.35**
Hostility	0.81	0.56	(0.60)	1.0	1.4	1.6	0.54	(0.64)	1.0	1.6	2.0	0.03
Paranoid Ideation	0.80	0.71	(0.73)	1.4	1.9	2.4	0.56	(0.61)	1.2	1.4	1.7	3.40
Sleep Disorders	0.77	1.14	(1.12)	1.8	3.0	3.1	0.88	(0.88)	1.5	2.4	2.7	7.23**
Global Severity Index	0.97	0.70	(0.53)	1.1	1.5	2.0	0.53	(0.47)	0.9	1.1	1.6	5.81*
Positive Symptom Distress Index		1.65	(0.49)	2.1	2.4	2.7	1.48	(0.38)	1.8	2.0	2.3	8.26**
Positive Symptoms Total		34.9	(20.5)	49	68	76	29.9	(20.6)	48	61	69	3.49

Significant effect sizes 0.02 ≤ η^2^ ≤ 0.03. Normative tables are available at request. Scales: N 309–332; Global indices 274–276

*p ≤ 0.01, **p ≤ 0.001

When interviewed for psychological and psychiatric evaluations, BS candidates also reported their own ideal weight, which was likely to be different from the BMI of 25 expected by medical criteria after surgery; all drugs they were currently using, including psychotropic medications, were recorded as well. For the present study, use of antidepressants and use of anxiolytics were treated as dichotomous variables (yes/no).

#### Outcome variable

In longitudinal analyses, the percentage of expected BMI loss was the outcome variable, calculated as follows:

$$\% {\text{EBMIL}} = \left[ {\left( {{\text{Preoperative\,BMI}} - {\text{Current\,BMI}}} \right)/\left( {{\text{Preoperative\,BMI}} - 25} \right)} \right] \times 100$$

where 25 represents the upper threshold of the healthy weight range; %EBMIL values ≥ 50 1 year after surgery are regarded as successful.

### Analysis

In the present study, we included all interviewed obese patients when we analyzed pre-treatment data, but BS patients only when we examined postoperative weight loss across 2 years. In addition to representing non-surgical patients, in the present sample 81 per cent of patients with intragastric balloons showed up for a 3–6-month follow-up, but only 28 per cent did for 12-month follow-up.

At the baseline (t0_INT_), we examined descriptive statistics and between-group (gender and type of intervention) differences (ANOVA) for each quantitative study variable and explored the factor structure (principal axis factoring, varimax rotation) of the SCL-90 items. We then inspected longitudinal cross-lagged regression patterns to predict weight loss (%EBMIL) at 3–6-month, 12- and 24-month follow-ups from preintervention SCL-90 profiles. Specifically, we selected significant SCL-90 original scales as well as empirical factors via an automatic stepwise procedure, after systematically equating participants for BMI at surgery (t0_SUR_), perceived ideal BMI at interview, gender, age, time occurred between interview and intervention, and use of psychotropic medications, further including among predictors %EBMIL observed at the follow-up occasion(s) prior to the %EBMIL outcome, to adjust for weight loss already achieved.

## Results

### Actual and perceived ideal BMI before the intervention

Self-reported data were collected at interview (t0_INT_), but patients underwent the intervention 10.8 ± 5.5 months later (t0_SUR_); in the meantime, actual BMI did not statistically change (test–retest *r* = 0.89, *p* ≤ 0.001; BMI increase = 0.32, *p* > 0.05). Notably, such an interval implies that the time period occurred between self-reports and a post-surgery follow-up occasion on average was 11 months longer than the interval between surgery and the follow-up occasion.

At interview, patients showed interindividual differences in perceiving their post-intervention ideal weight, with a mean ideal BMI of 27.0 ± 3.2, with men (28.5 ± 2.9) generally reporting a higher ideal BMI compared with women (26.4 ± 2.9, *p* ≤ 0.001); more obese patients self-reported higher post-surgery ideal BMI (r = 0.41, *p* ≤ 0.001). No differences in actual BMI were observed between patients who used vs. did not psychotropic medications, whereas patients taking an antidepressant drug (*N* = 47) referred slightly lower ideal BMI values (26.1 ± 2.2) compared with patients who were not (27.2 ± 3.2, *p* ≤ 0.05).

### Normalization of SCL-90 scores

SCL-90 raw scores generally deviated from normality. Therefore, we preliminarily converted the raw scores into normalized T-scores, after controlling for gender differences. In the present work, subsequent analyzes were performed on the SCL-90 T-scores. Table 1 presents mean scores for SCL-90 scales and global indices and the values for percentile ranks 50, 80, 90, and 95 for the present sample, separately for women and men. The results in Table 1 show that the conventional clinical cutoff value of 0.9 corresponds approximately to percentile rank 80 for the GSI score, but is generally misleading for the clinical scales [see also 35].

### SCL-90 factor structure

When the items were subjected to exploratory factor analysis, the parallel analysis indicated an 8-factor solution (48.1% of accounted variance); the Root Mean Square Error of Approximation (RMSEA) as a fit index was equal to 0.05 (90% CI 0.05–0.06) and indicated a good fit of the model to the empirical data. The eight factors are listed in Table 2. They only partially corresponded to the expected structure of the SCL-90 questionnaire, nevertheless they were conceptually coherent and could be interpreted as follows: (1) Relational Distress, encompassing items from different SCL-90 scales and expressing interpersonal sensitivity, suspiciousness, and insecurity; (2) Somatization, including physical complaints, sleep problems, and depression-related items expressing low energy; 3) Dysphoria, combining mostly nervousness, apprehension, and feeling down; (4) Generalized Anxiety, experiencing intense feelings of fear; (5) Aggressive Hostility, representing hostile feelings and urges to externalize anger; (6) Depressive Ideation, feelings of hopelessness; (7) Phobias, a mixed factor, where specific phobias predominated; (8) low Concentration as a subcomponent of the Obsession–Compulsion scale.

**Table 2:** Exploratory varimax rotated 8-factor solution of the SCL-90 items

Item	1	2	3	4	5	6	7	8
69. Feeling very self-conscious with others	**0.73**							
43. Feeling that you are watched or talked about by others	**0.70**							
61. Feeling uneasy when people are watching or talking about you	**0.65**							
41. Feeling inferior to others	**0.63**		0.33					
58. Heavy feelings in your arms or legs		**0.70**						
56. Feeling weak in parts of your body		**0.69**						
42. Soreness of your muscles		**0.68**						
27. Pains in lower back		**0.56**						
30, Feeling blue		0.35	**0.63**					
29. Feeling lonely	0.32		**0.57**					
57. Feeling tense or keyed up		0.32	**0.55**					
31. Worrying too much about things	0.35		**0.53**					
72. Spells of terror or panic				**0.62**				
25. Feeling afraid to go out of your house alone				**0.61**				
82. Feeling afraid you will faint in public				**0.60**				
23. Suddenly scared for no reason	0.30			**0.53**		0.34		
67. Having urges to break or smash things					**0.64**			
74. Getting into frequent arguments					**0.59**			
81. Shouting or throwing things					**0.58**	0.36		
24. Temper outbursts that you could not control					**0.57**	0.30		
15. Thoughts of ending your life						**0.62**		
59. Thoughts of death or dying						**0.49**		
16. Hearing words that others do not hear						**0.46**		
51. Your mind going blank		0.32				**0.44**		
75. Feeling nervous when you are left alone							**0.53**	
50. Having to avoid certain things, places, or activities because they frighten you					0.34		**0.48**	
80. Feeling that familiar things are strange or unreal							**0.44**	
13. Feeling afraid in open spaces or on the streets	0.31						**0.43**	
9. Trouble remembering things								**0.56**
55. Trouble concentrating								**0.55**
38. Having to do things very slowly to insure correctness	0.30	0.31						**0.36**
10. Worried about sloppiness or carelessness								**0.33**
% accounted variance (after rotation)	*9.92*	*8.48*	*7.32*	*5.47*	*5,45*	*4.69*	*4.28*	*2.52*

Communalities 0.33 ≤ *h*^*2*^ ≤ 0.73. Factor loadings ≥ 0.30 are reported; Primary factor loadings are in bold. *N* = 275

Table 3 presents the concurrent unique associations between each of the eight empirical factors and the original SCL-90 scales, after conducting multiple regression analyses, with the SCL-90 scales simultaneously entered as predictors of each SCL-90 empirical factor. A horizontal reading of Table 3 evidences that the SCL-90 Somatization, Obsessiveness, Interpersonal Sensitivity, and Aggressive Hostility scales were mostly represented by a single factor; conversely, the remaining scales were distributed across different empirical factors and recombined with each other. For example, the SCL-90 Phobic Anxiety scale split up into 3 main empirical factors, namely, Dysphoria, Phobias, and Generalized Anxiety. No empirical dimension matched Psychoticism.

**Table 3:** Predicting SCL-90 factor scores from SCL-90 scale scores

	1	2	3	4	5	6	7	8
Somatization		0.59*	− 0.29*			− 0.11		
Obsession–compulsion								0.67*
Depression			0.33*	− 0.24*		0.27*		
Anxiety			0.18*	0.14			0.12	− 0.15*
Phobic anxiety			− 0.30*	0.37*		0.11	0.54*	
Psychoticism								− 0.14*
Interpersonal sensitivity	0.47*			− 0.17*	− 0.13*		− 0.19*	
Hostility	− 0.24*			0.11	0.75*		− 0.07	
Paranoid ideation	− 0.16*	− 0.13*		0.20*	0.10	− 0.30*		
Sleep Disorders		0.25*	0.16*	0.22*		− 0.27*	− 0.33*	
*R*^*2*^_Adj_	0.70*	0.78*	0.58*	0.29*	0.70*	0.24*	0.50*	0.51*

*N* = 256, after excluding univariate outliers with factor z-scores > │3│. Semi-partial correlations ≥ 0.10 are presented. Factor labels: 1. Relational Distress, 2. Somatization, 3. Dysphoria, 4. Generalized Anxiety, 5. Aggressive Hostility, 6. Depressive Ideation, 7. Phobias, 8. low Concentration. *p ≤ 0.001

Significant simple correlations (0.16 ≤ *r* ≤ 0.19, *p* ≤ 0.01) were observed between use of antidepressants and both SCL-90 Depression, Somatization, Anxiety scales and SCL-90 Dysphoria and Somatization empirical factors; though small in magnitude, they support external validity of SCL-90 original as well as empirical scale scores against an objective criterion.

### Concurrent associations between SCL-90 and BMI

In BS patients, no significant simple correlation was observed between pre-surgery BMI (t0_INT_) and SCL-90 scales or global indices. After adjusting for gender, age, and use of antidepressants and anxiolytics (*R*^2^ = 0.03, *p* ≤ 0.05, but no significant unique associations), hierarchical regression analysis (stepwise procedure) revealed that SCL-90 Anxiety (*β* = − 0.27, *p* ≤ 0.01) and Paranoid Ideation (*β* = 0.26, *p* ≤ 0.01) accounted for an additional and significant variance proportion of BMI at interview (∆*R*^2^ = 0.05, *p* ≤ 0.01); moreover, when SCL-90 empirical factors were added in the regression model, Dysphoria accounted for a further small variance proportion (∆*R*^2^ = 0.02, *p* ≤ 0.05).

### BMI loss trends

**Fig. 1:**
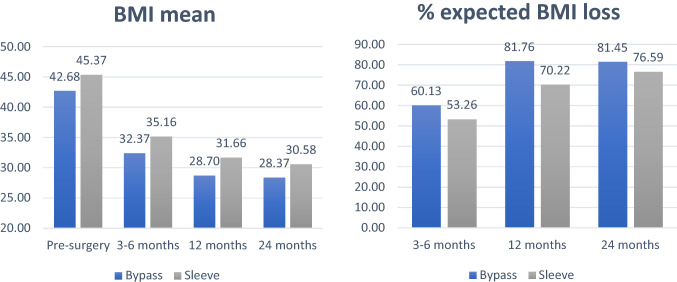
BMI and %EBMIL mean levels across time

Figure 1 illustrates BMI and %EBMIL trends across time for BS patients only.^1^ Over 12 months, ANOVA for repeated measures (with 6 months occurred between occasions) indicated a quadratic trend (p ≤ 0.001), with a larger decrease within the first semester. Over 24 months (with 12 months occurred between occasions), ANOVA showed a quadratic trend (*p* ≤ 0.001) as well. Overall, BS patients generally lost most of their weight during the first year—first semester especially—and successfully maintained it in the second year after surgery, with %EBMIL > 75.

### Predicting BMI loss after surgery over different timepoints

When we inspected how SCL-90 scales and factors predict weight loss (%EBMIL) 3–6 months after surgery, adjusting for gender, age, t0_SUR_ actual BMI, t0_INT_ ideal BMI, and use of antidepressants (use of anxiolytics and time occurred between interview and intervention were not significant in any analysis we performed and were included no longer among predictors), we found that only pre-surgery actual BMI accounted for a significant unique variance proportion equal to 16 per cent (*β* = − 0.42, *p* ≤ 0.001) of the outcome.^2^

The empirical factor of Relational Distress uniquely predicted (*β* = 0.23, ∆*R*^2^ = 0.05, *p* ≤ 0.01) %EBMIL at 12 months, after controlling for %EBMIL at 3–6 months (*β* = 0.62, *R*^2^ = 0.42, *p* ≤ 0.001, *N* = 92) as well, that is, higher Relational Distress anticipated additional weight loss from 3 to 6 months to 1 year after intervention; any other variable was not significant in the prediction model. Over a longer time period, i.e., 24 months after operation, SCL-90 factors of Relational Distress (*β* = − 0.25, *p* ≤ 0.01), Generalized Anxiety (*β* = − 0.23, *p* ≤ 0.01), and Phobias (*β* = − 0.20, *p* ≤ 0.05) predicted a significant variance proportion in post-surgery weight loss (∆*R*^2^ = 0.16, *p* ≤ 0.001), in addition to pre-surgery lower perceived ideal BMI (*β* = − 0.29, *p* ≤ 0.01), no use of antidepressants (*β* = − 0.29, *p* ≤ 0.01), and higher weight loss already achieved (1-year % EBMIL, *β* = 0.73, *p* ≤ 0.001), adjusting for gender, age, initial BMI and %EBMIL at 3–6 months after surgery.

**Fig. 2:**
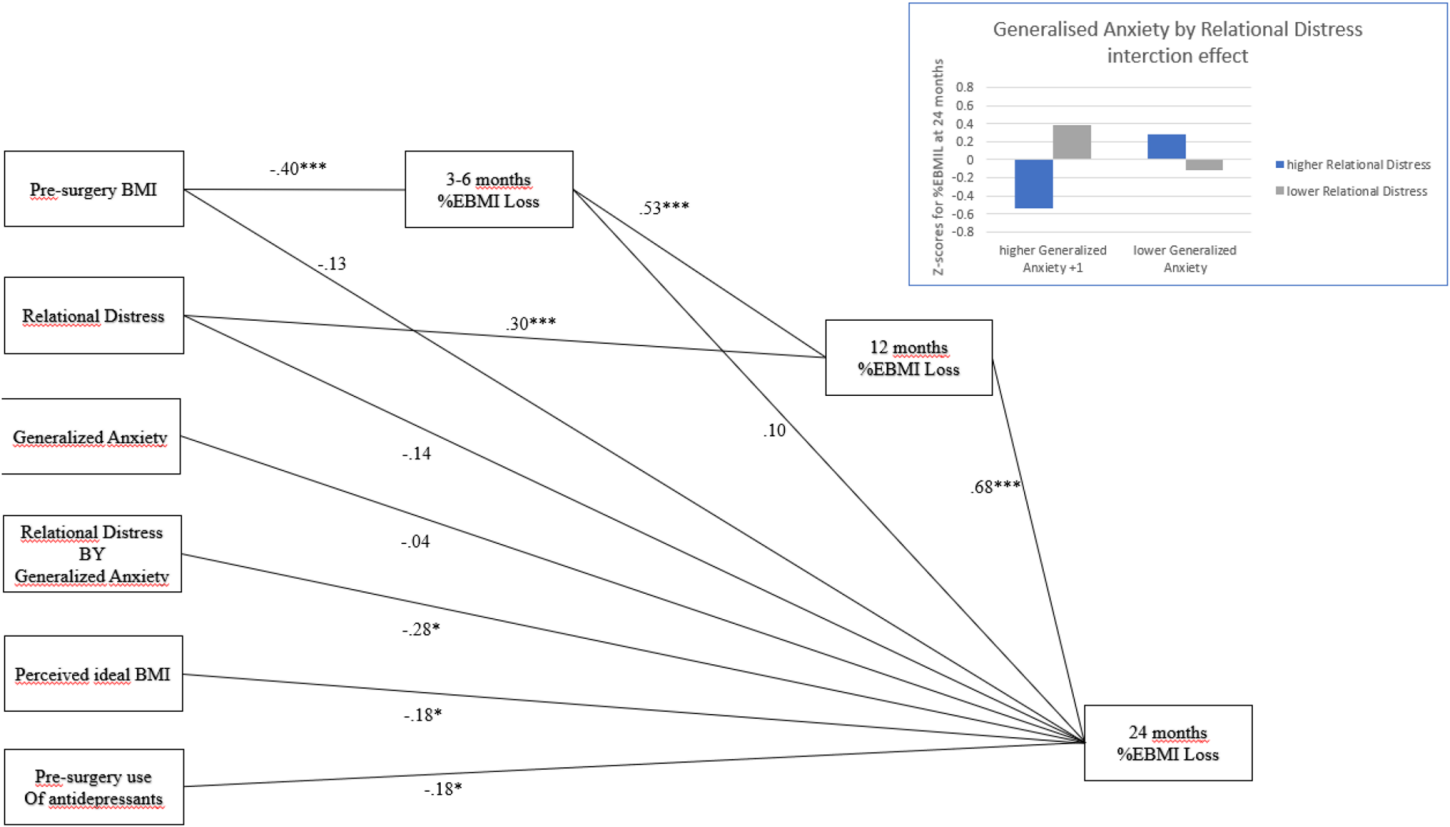
Paths directly linking pre-surgery variables to weight loss as %EBMIL across follow-up occasions. Beta weights are presented; results were adjusted for gender (*β* = − 0.10, p* >* .05), and Phobias factor scores (*β* = − 0.14, *p* > 0.05). The figure in the box illustrated the interaction effect between generalized anxiety and relational distress (higher =  + 1 sd, lower =− 1 sd). %EBMIL = percentage of BMIL loss. *p ≤ 0.05, ** *p* ≤ 0.01, ***p ≤ 0.001

Finally, we inspected a provisional mediation model, to examine the hypotheses suggested by the above results, that is, (a) pre-surgery BMI had an indirect effect on 24-month outcome via its effect on weight loss across the different timepoints, and (b) Relational Distress had an indirect effect on outcome at 24 months via its effect on BMI loss at 12 months, equating participants for gender, perceived ideal BMI, Phobias factor, and use of antidepressants. The results from path analysis (Fig. 2) supported both the expected cascade effect of t0_SUR_ BMI on weight loss across the measurement timepoints, with a significant indirect effect of t0_SUR_ BMI on 2-year %EBMIL (*β* = − 0.14, *p* ≤ 0.01) as well as an indirect effect of Relational Distress on %EBMIL outcome (*β* = 0.20, *p* ≤ 0.01). The full model additionally revealed a significant long-term effect of a patient’s perceived ideal BMI (*β* = − 0.18, *p* ≤ 0.05), use of antidepressants (*β* = − 0.18, *p* ≤ 0.05), and that Relational Distress and Generalized Anxiety synergically interacted (*β* = − 0.33, *p* ≤ 0.01), that is, higher Relational Distress levels amplified the negative impact of higher Generalized Anxiety levels, whereas higher relational uneasiness remained protective against weight regain if combined with low anxiety.

## Discussion

The present longitudinal study examined how pre-surgery psychological health, in the form of SCL-90 profiles, helps predict post-surgery weight loss success in bariatric patients. The main finding is that the SCL-90 empirical factor scores, but not the original scales or global indices, significantly predicted postoperative outcome in bariatric patients. Of note, SCL-90 self-reports had been collected on average 11 months before surgery, meaning they predicted BS outcome up to 3 years later. This finding opens new perspectives on the SCL-90, a widely used screening self-report instrument for psychopathological disorders, which, however, provides inconsistent and weak longitudinal evidence in establishing its predictive validity in clinical contexts [20, 25].

No previous study has examined the structure of the SCL-90 items in candidates for bariatric surgery, though deviations from the theoretical factor structure are not an exception [28, 29]. In fact, our results yielded an interpretable 8-factor solution that only partially replicated the theoretical structure, but resembled the structure Prunas et al. [28] found in a large sample of non-clinical adults. Such a bottom-up approach organized the SCL-90 items into meaningful domains for bariatric candidates, thereby hypothetically helping anticipate post-intervention success.

In fact, the longitudinal results of our study showed that the traditional SCL-90 scales and global indices did not predict BS outcome at any follow-up point, although concurrently the Anxiety and Paranoid Ideation scales correlated with BMI at interview [8–10]. Conversely, the empirical factors of Relational Distress and Generalized Anxiety predicted BMI loss across years. Specifically, the results revealed that pre-intervention Relational Distress was directly protective against weight regain 12 months after intervention.

Moreover, Relational Distress had an effect on 2-year BS outcome indirectly (via its positive effect on 1-year weight loss) as well as in interaction with Generalized Anxiety: the combination of high anxiety and high relational uneasiness had a detrimental effect on the surgical outcome. In general, the finding that emotional instability, i.e., dysphoria, depression, and anxiety, or relational uneasiness anticipate weight loss is not fully consistent in empirical studies [11, 21]. Nonetheless, our study strengthens the evidence that attention should be paid to pre-surgery social discomfort especially when combined with anxiety, to achieve better outcomes after surgery [38]. Moreover, the long-term effect of the preintervention use of antidepressant on BS success further underlines that emotionally fragile BS patients, though clinically healthy, need more support on the long run.

The strongest predictor of bariatric surgery success was BMI before surgery, with a chain effect across time. Indeed, in the present study, the longitudinal trend in BMI loss was quadratic, with greater weight loss in the first semester and progressively smaller and smaller weight loss up to 2 years. Such a trajectory is fully consistent with the literature [17, 39] and further highlights the importance of helping patients cope with the difficulties they face during the first postoperative semester, because initial outcomes are predictive of later success.

Finally, our study revealed that a patient’s perceived ideal post-surgery weight may also play a role in predicting bariatric success. Studies have documented how obese patients generally overestimate their target ideal weight compared with an expected optimal weight, and such misperception is considered critical for obesity prevention programs [34]. The current study was the first to show that a non-oversized body image may represent a protective factor in the long term.

## Clinical implications

Overall, a patient evaluation procedure for bariatric surgery is complex, time-consuming, and requires a multidisciplinary team to select individuals who could mostly benefit from a costly procedure that may lead to severe complications. Psychological and psychiatric assessment procedures also are demanding, and include the administration of several quantitative instruments as well as individual clinical interviews. They allow to select eligible candidates for bariatric surgery, but currently their long-term predictive validity remains under debate. The main clinical implication of the present findings is that pre-surgery SCL-90 profiles help identify patients with greater postoperative success at different timepoints, provided that empirical factor scores are inspected. Thus, if the present results are replicated in independent samples, this single screening instrument could be routinely applied in clinical practice to evaluate the mental health of a BS candidate and anticipate her or his long-term (un)success. In fact, most of patients lose a large amount of weight during the first few months after intervention, but it becomes difficult for some of them to keep on losing weight until they reach their target weight and maintain it over longer periods. Our findings suggest that pre-surgery SCL-90 profiles combining high relational distress and high generalized anxiety warn against the risk of a poorer success in the long run and thereby suggest that patients with such preintervention profiles, though clinically subthreshold, could benefit from a regular monitoring and psychological treatment both before and after intervention. Finally, our findings also indicate that more attention should be provided to a patient’s perceived ideal weight and obesity prevention programs could help them targeting a healthier postoperative weight and body silhouettes [34].

## Strengths, limits, and future research

This longitudinal study suggests to investigate pre-surgery psychological distress and health from a bottom-up approach in bariatric patients, when assessment instruments developed for different target populations are applied. In fact, longitudinal findings evidenced that the SCL-90 empirical factors outperformed the SCL-90 scales and indices in predicting BS outcome. However, there is need for cross-validating the present findings and several limitations have to be acknowledged. First, our data set did not include patients who dropped out before or after bariatric surgery nor patients with medical or psychiatric comorbidities and thereby no longer eligible for bariatric surgery. Second, though we controlled our results for use of patients’ psychotropic medications, we didn’t adjust the present results for lifetime comorbid psychiatric or eating disorders. In fact, lifetime Axis I psychiatric disorders as well as disordered eating behaviors could affect bariatric surgery success, even if the psychiatric condition is in remission before surgery. Third, we examined a questionnaire in its form of self-report, although all patients also underwent a psychiatric evaluation which consisted in a face-to-face clinical interview, which allowed psychiatrists to refine their diagnosis on the presence of a current and/or lifetime psychiatric disorder and possibly exclude patients with unstable and not adequately treated mental disorders. Therefore, we acknowledge the use of self-reported questionnaires only in predicting bariatric success as a possible limitation. Finally, we acknowledge that there is also need to extend the present results by including post-intervention psychological distress, so as to better understand the interplay between weight changes and SCL-90 variables. In brief, BS outcome depends on several medical, psychological and relational determinants, but we focused our attention on a single piece of a large picture.

## What is already known on this subject

Several studies have investigated the same question, with inconsistent results; only pre-intervention weight represents a significant predictor of post-surgery success, and we contributed to establish its clinical relevance.

## What this study adds

This study reveals that a re-combination of the SCL-90 items into empirical domains, presumably meaningful for BS candidates, helps predict BS success on the long run especially; relational uneasiness deserves attention, because of its direct, indirect and moderated (in combination with anxiety) effects on weight loss over a long time period; initial weight loss has a cascade effect across time; a patient’s perceived ideal weight impact postoperative weight loss, though underlying mechanisms need to be investigated.

## Data Availability

Data analysed for the current study are available from the corresponding author on request.

## Footnotes

1 A first comparison included both BS patients and patients with intragastric balloon, over 3–6 months. Results from mixed design ANOVA showed a significant interaction 2 (pre- vs. post-treatment BMI) × 2 (surgical vs. endoscopic treatment) effect (*p* ≤ .001, partial η^2^ = .24), revealing a poorer BMI loss in patients with intragastric balloon (-4.6) compared with BS patients (− 9.8).

2 When patients with intragastric balloon were included as well, the results showed that initial higher SCL-90 Generalized Anxiety factor scores predicted higher %EBMIL levels (∆R^2^ = .05, *p*  ≤  .05), but the interaction effect Generalized Anxiety by type of intervention (surgical vs. endoscopic intervention) was not significant (*p*  >  .05).
